# Comprehensive analysis of *LILR* family genes expression and tumour‐infiltrating immune cells in early‐stage pancreatic ductal adenocarcinoma

**DOI:** 10.1049/syb2.12058

**Published:** 2023-02-07

**Authors:** Qiang Gao, Shutian Mo, Chuangye Han, Xiwen Liao, Chengkun Yang, Xiangkun Wang, Tianyi Liang, Yongfei He, Zijun Chen, Guangzhi Zhu, Hao Su, Xinping Ye, Tao Peng

**Affiliations:** ^1^ Department of Hepatobiliary Surgery The First Affiliated Hospital of Guangxi Medical University Nanning China; ^2^ Guangxi Key Laboratory of Enhanced Recovery After Surgery for Gastrointestinal Cancer Nanning China

**Keywords:** bioinformatics, cancer, surgery

## Abstract

Leucocyte immunoglobulin‐like receptors (LILRs) are closely related to tumourigenesis, but their clinical value in early‐stage pancreatic ductal adenocarcinoma (PDAC) after pancreaticoduodenectomy remains unknown. Kaplan–Meier and Cox proportional hazards regression models is used to investigate the association between LILR expression and prognosis in tumour biopsies and peripheral blood mononuclear cells. Risk score was calculated for each patient based on the prognostic model. DAVID, STRING, GeneMANIA, and GSEA were used to conduct pathway and functional analyses. The CIBERSORT algorithm is used to analyse tumour‐infiltrating immune cells. Survival analysis showed that high levels of *LILRA4* (*p* = 0.006) and *LILRB4* (*p* = 0.04) were significantly associated with better overall survival. High levels of *LILRA2* (*p* = 0.008) and *LILRB4* (*p* = 0.038) were significantly associated with better relapse‐free survival. JAK‐STAT signalling pathway, regulation of T cell activation, regulation of the immune effector process, and tumour necrosis factor superfamily cytokine production were involved in molecular mechanisms that affected poor prognoses in the high‐risk group in GSEA. CIBERSORT demonstrated that the high‐risk group had significantly higher infiltrating fraction of memory‐activated CD4 T cells and activated NK cells and lower fraction of resting dendritic cells and neutrophils. *LILRB4* plays crucial roles in affecting the clinical outcomes of early‐stage PDAC.

## Graphical abstract

*LILRB4* play crucial roles in affecting the clinical outcomes of early‐stage PDAC through JAK‐STAT signalling pathway, immune‐related signalling pathways, and tumour‐infiltrating immune cells.

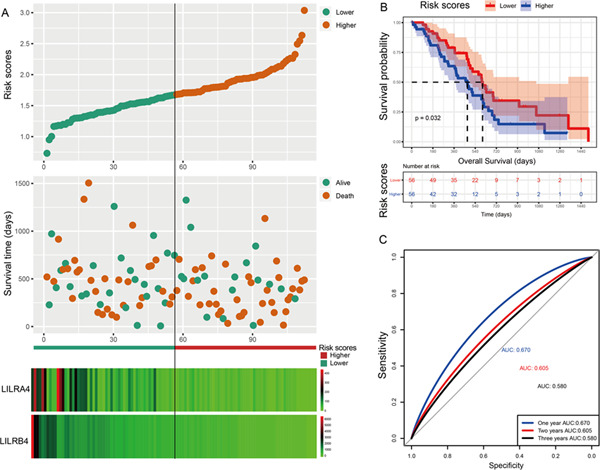

**Abbreviations**
DCs: dendritic cells
FDR: false discovery rate
GEO: gene expression omnibus
GO: gene ontology
GSEA: gene set enriched analysis
HLA‐G: human Leucocyte Antigen‐G
HR: hazard ratios
KEGG: Kyoto Encyclopaedia of Genes and Genomes
KIR: killer cell inhibitory receptor
KM: Kaplan‐Meier
LILRs: Leucocyte immunoglobulin‐like receptors
MSigDB: Molecular Signatures database
NK: natural killer
OS: Overall survival
PBMCs: peripheral blood mononuclear cells
PDAC: pancreatic ductal adenocarcinoma
RFS: relapse‐free survival
ROC: receiver operating characteristic
TCGA: The Cancer Genome Atlas
TIICs: tumour‐infiltrating immune cells

## 1 BACKGROUND

According to global cancer statistics, in 2018, about 458,918 new cases of pancreatic cancer occurred, and the number of deaths was about 432,242 worldwide, indicating similar morbidity and mortality rates [1]. Pancreatic ductal adenocarcinoma (PDAC) is the main pathological type of pancreatic cancer. It has the characteristics of high malignancy and rapid progression, resulting in poor clinical prognosis [2]. In recent years, with the improvement of PDAC treatment strategies and the emergence of new treatment methods, such as immunotherapy and neoadjuvant chemotherapy, the clinical outcomes of PDAC have improved to some extent, but the challenge is that the 5‐year overall survival (OS) rate is still less than 10% in all stages of PDAC and it still has the worst prognosis among all cancers [3, 4]. Although the 5‐year OS of patients with pancreatic cancer has risen from 2.5% in 1970 to 9% in 2019, there is still a large gap in the improvement of the clinical outcomes of other tumour types, such as breast and colorectal cancers [3, 4, 5]. Lack of biological indicators for early effective screening strategies and novel therapies in PDAC are the main reasons for this discrepancy [6, 7]. Current studies have shown that tumour markers, such as various commonly used carcinoembryonic antigens and carbohydrate antigen 199, are not effective in diagnosing early PDAC due to their low sensitivity [7]. However, early detection strategies involving endoscopic ultrasound and magnetic resonance imaging have not been validated in randomised trials in high‐risk PDAC patients [4]. Therefore, identifying effective screening indicators and treatment‐related targets is crucial to improve the prognosis of pancreatic cancer.

PDAC is similar to most adenocarcinomas, with a massive fibrotic stroma, which plays an important role in the local inflammatory microenvironment of tumours [8, 9, 10]. The microenvironment of PDAC consists of abundant deposition of the extracellular matrix, low vessel density, cancer‐associated fibroblasts, and immune/inflammatory cells, which are closely related to tumour growth and progression and the infiltration of immune cells, similar to other tumours [2, 11, 12, 13, 14, 15]. Immune cells may be found in solid tumours, and different types of immune cells have different effects on the clinical prognosis of tumours [16, 17]. In particular, increased number of dendritic cells (DCs) is associated with improved prognosis in various types of human cancers. DC maturation is a prognostic indicator; moreover, chronic inflammation and presence of M2 macrophages facilitate tumour growth and spread [18, 19].

*Leucocyte immunoglobulin‐like receptors* (LILRs) are a family of receptors with extracellular immunoglobulin domains; the gene coding region is located on the chromosome region 19q13.4, also known as CD85, ILT, and LIR, which have immunomodulatory effects on a variety of immune cells [20, 21]. LILRs include subfamily A and subfamily B (*LILRA1–6* and *LIRB1–5*, respectively). The LILR receptor in the A subfamily is an activation receptor that contains tyrosine‐based immune receptor activation motifs (ITAMs), while the LIR receptor in the B subfamily contains multiple tyrosine‐based cytoplasmic immune receptor inhibition motifs (ITIMs) [22, 23]. LILRs are related to the human killer cell inhibitory receptor (KIR) family. Both have similar Ig‐like structures and cytoplasmic signal domains. Although the expression of KIR is limited to natural killer (NK) cells, LILRs are found in a variety of cells, including NK, T lymphocytes, B lymphocytes, and myeloid cells (monocytes, macrophages, dendritic cells, and granulocytes [24]). The transmembrane domain of the LILRA receptor contains a charged arginine or lysine residue associated with the FcR ligand containing (ITAXI/Lx 6–12 YxxI/L) ITAM [25]). ITAM activation recruits Syk/ZAP70 family kinases to drive downstream activation pathways, which are important for immunity [26]. By contrast, the LILRB receptor contains the cytoplasmic (S/I/V/LxYxxI/V/L) ITIM domain, which recruits the phosphatase SHP1/SHP2/SHIP containing the Src homology 2 domains, thereby inhibiting the immune signalling cascade. SHP/SHIP phosphatase activity is essential to maintain immune homoeostasis [27].

LILRAs and LILRBs belong to the LILRs group, and the main function of LILRAs involves immune activation, while the role of LILRBs is immune suppression [28, 29, 30]. A study on oestrogen receptor‐positive breast cancer reported that after receiving neoadjuvant endocrine therapy, compared with patients with low expression of the LILRA2 gene in the tumour biopsies, patients with high *LILRA2* expression showed significant tumour shrinkage, which was beneficial to breast‐conserving surgery [31]. Lu et al. found that the Semaphorin‐4A gene stimulated the CD4+ T cells and regulated Th2 T cell differentiation by binding to *LILRA2*, thereby initiating an immune response [32]. A previous study reported that *LILRA4* was also expressed in cancer cells, which was associated with impairment of plasmacytoid dendritic cells (pDCs) in the microenvironment of cancers [33]. A genome‐wide analysis of copy number variations in ovarian cancer has shown that duplicate mutations in *LILRA6* were associated with susceptibility to high‐grade serous ovarian cancer [34].

Human Leucocyte Antigen‐G (HLA‐G) positive expression in gastric cancer patients indicated a poor prognosis, and its possible mechanism may be that HLA‐G combined with *LILRB1* inhibited NK cell proliferation and function [35]. A series of studies have shown that in a variety of tumours, such as colon cancer, non‐small cell lung cancer, and hepatocellular carcinoma, overexpressed *LILRB2* was associated with a poor prognosis [36, 37, 38, 39, 40, 41]. *LILRB4*, which is also an immunosuppressive receptor, is similar to *LILRB3*, so it can interact with HLA‐G to inhibit the activation of immune cells, which mainly include NK and T cells [42, 43]. High *LILRB4* expression has also been reported to be associated with tumour progression and poor prognosis. *LILRB4* was associated with impaired T cell responses in pancreatic cancer, and antagonistic *LILRB4* was the key to successful immunotherapy [44, 45]. In leukaemia, tumour cells disable immune checkpoint blockade therapy through the *LILRB4* signalling, and blocking *LILRB4* can prevent the development and metastasis of tumour cells. The potential mechanism is that *LILRB4* changes the tumour microenvironment, resulting in immune suppression [46, 47]. In colorectal cancer, the *LILRB4* gene was found to be highly expressed in cancer tissue, and the expression level of the *LILRB4* gene was negatively correlated with the density of CD45RO + T cells in the cancer tissue, and high *LILRB4* gene expression was a biomarker of poor clinical prognosis for colorectal cancer [48]. Moreover, in gastric cancer, *LILRB4* was significantly related to the pathological grade [49]. Therefore, we know that LILR family genes play an important role in tumourigenesis, development, and clinical prognosis.

However, the effect of LILRs on clinical outcomes in patients with PDAC remains unknown. This study aimed to investigate the association between LILRs gene expression and prognosis and reveal possible mechanisms in pathway enrichment and tumour immune cell infiltration in early‐stage PDAC.

## 2 MATERIALS AND METHODS

### 2.1 Data mining and processing

The gene expression profiles and clinical information of PDAC were obtained from The Cancer Genome Atlas (TCGA, data release 21.0, December 10, 2019) and normalised by the ‘DESeq’ and ‘Limma’ package in R (version 3.6.1; www‐project.org) [50]. The inclusion criteria for cases in this study were as follows: (i) patients with pancreaticoduodenectomy and pathologically confirmed as PDAC, (ii) according to the Seventh American Joint Committee on Cancer, patients with postoperative specimen pathological stage were stage I or II, and pathology stages I and II were defined as early‐stage PDAC, and (iii) patients with complete clinical prognosis data. To further characterise the expression levels of LILRs between PDAC patients and healthy control patients in the peripheral blood mononuclear cells (PBMCs), GSE74629 and GSE49641 were obtained from the gene expression omnibus (GEO; http://www.ncbi.nlm.nih.gov/geo) database. GSE55643 was used to verify differences in gene expression levels. Data from the GEO database were normalised by the ‘normalizeBetweenArrays’ function of ‘Limma’ package in R.

### 2.2 Bioinformatics and correlation analysis of LILR genes

First, we analysed the differences in LILR gene expression between pancreatic cancer and adjacent tissues and constructed violin plots using the ‘wilcox.test’ function of ‘vioplot’ package in R. We then used the ‘corrplot’ package in R to conduct Pearson's correlation analyses between LILR genes and then constructed corresponding correlation plots. We used the online tool DAVID Bioinformatics Resources (version 6.8, https://david.ncifcrf.gov) to conduct functional annotation of gene ontology (GO) terms and enrichment of the Kyoto Encyclopaedia of Genes and Genomes (KEGG) for all LILR genes. All 10 genes from the LILR gene family were included in this analysis. [51, 52]. Finally, we analysed the interrelationships of gene–gene and protein–protein interactions between LILRs genes using online analysis tools: GeneMANIA([53]) (http://genemania.org) and STRING (https://string‐db.org) [54].

### 2.3 Survival analysis and prognostic model construction

Using the median expression of each LILR gene as the cut‐off point, we defined the high‐ and low‐expression groups. Kaplan–Meier analysis with the log‐rank test was used as a univariate analysis to assess the association between clinical factors, gene expression, and clinical outcomes, including OS and relapse‐free survival (RFS). Clinical factors with *p*‐values of the log‐rank test less than 0.05 were used as corrective factors when constructing the Cox proportional hazards regression model of each LILR gene. Then, the genes that were significantly associated with clinical outcomes were recruited to construct a prognostic model. The formula of risk scores was as follows:

$$Risk\;score={expression\;of\;gene}_{1}\times \beta_{1}+{expression\;of\;gene}_{2}\times \beta_{2}+\text{…}\;{expression\;of\;gene}_{n}\times \beta_{n}$$

where *β* is the regression coefficient from the multivariate Cox proportional regression model of individual genes. We used median risk scores as the cut‐off point and divided the patients with PDAC into higher and lower risk groups. A receiver operating characteristic (ROC) curve was used to evaluate the predictive power of the prognostic model, which was conducted using the ‘roc’ function of ‘ROC’ package in R [55]. Subsequently, we combined the risk score with clinical factors of *p*‐values less than 0.05 for a nomogram analysis, which was also completed using the ‘rms’ and ‘survival’ packages of R[56]. Afterwards, we analysed differences in expressions of prognosis‐related genes between PDAC patients and healthy control patients in the peripheral blood and PBMCs and visualised the results using the ‘ggplot’ function and *t*‐test of ‘ggplot2’ package in R. ROC curves were constructed to show the diagnostic efficiency of prognosis‐related genes in the diagnosis of PDAC by using the ‘pROC’ package in R.

To verify the stability of the model, we used the k‐fold method that was used for cross‐validation of the model, ‘caret’ package to create cross‐validation data set, and ‘survivalROC’ package to calculate AUC values.

### 2.4 GSEA

After constructing the prognostic model, we found that compared with the lower risk group, the higher risk group had a worse clinical prognosis. To investigate the potential molecular mechanism of adverse prognosis in the higher risk group, we performed GSEA between both groups [57]. The Molecular Signatures database (MSigDB) C2 and C5 gene sets were used in the GSEA. The C2 gene set mainly included KEGG and related signalling pathway analysis, while the C5 gene set mainly included GO enrichment analysis. The selection criteria for statistically significant gene sets were a false discovery rate (FDR) less than 0.25 and a *p*‐value less than 0.05.

### 2.5 Analysis of tumour‐infiltrating immune cells

LILR genes were closely related to the regulation of immune cells, so we performed a tumour‐infiltrating immune cell (TIIC) analysis between the higher risk group and lower risk group. The CIBERSORT algorithm was used to perform tumour‐infiltrating immune cell analysis, which is a gene expression‐based deconvolution algorithm, which can measure 22 types of characteristic immune cell compositions in RNA mixtures from many tissues, including solid tumours [58]. The algorithm based on the mRNA expression sequence was conducted using R, and the set parameter was 1000 permutations.

### 2.6 Statistical analyses

All statistical analyses and plots were performed using SPSS 23.0 and R (version 3.6.1). A *p*‐value of less than 0.05 was considered statistically significant. Association analyses between clinical factors and gene expression and clinical outcomes were tested using Kaplan–Meier analysis and a Cox proportional hazard regression model, and hazard ratios (HRs) with 95% confidence intervals were used to describe the relative risks. A two‐sided *t*‐test was used to evaluate the differential expression analysis of LILR gene expression and the percentage of tumour‐infiltrating cells between the two groups. In GSEA, the Benjamini–Hochberg method was used to adjust multiple tests, which defined the meaning of FDR; in our study, FDR <0.25 was considered statistically significant.

## 3 RESULTS

### 3.1 Data processing

After standardised processing, 112 patients with PDAC and 31,777 genes who met the inclusion criteria from TCGA were enroled in our study. Clinical factors included age, sex, history of chronic pancreatitis and alcohol, tumour size, pathological stage, neoplasm histological grade, targeted molecular therapy, radiation therapy, residual resection, and information of clinical outcomes (Table S1). Overall survival data were available for all samples in the 112 PDAC patients, but 19 cases were missing in the relapse‐free survival data.

### 3.2 Bioinformatics and correlation analysis of LILR genes

**FIGURE 1:**
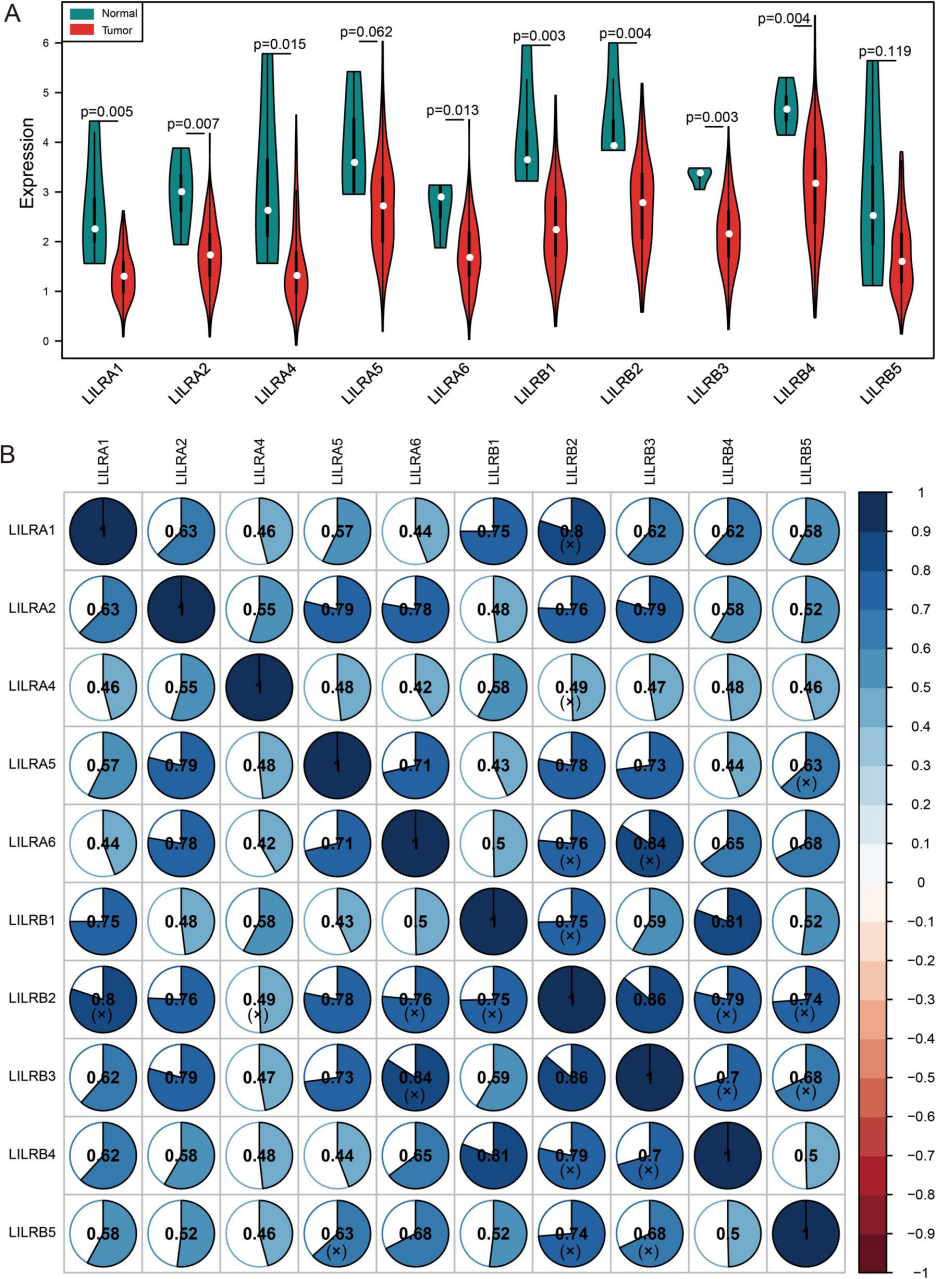
(a) Violin diagram of differential expression analysis and (b) correlation graph of Pearson's correlation analysis of LILRs genes; blue denotes a positive correlation, red denotes a negative correlation, and the shade of colour represents the size of the correlation coefficient. The (x) means that the results were not statistically significant.

We obtained 10 LILR gene expressions from the pancreatic cancer expression profile of TCGA. These included *LILRA1*, *LILRA2*, *LILRA4*, *LILRA5*, *LILRA6*, *LILRB1*, *LILRB2*, *LILRB3*, *LILRB4*, and *LILRB5* (Table S3). We performed a differential expression analysis of those genes between pancreatic cancer tissues and adjacent tissues. The results showed that compared with adjacent tissues, *LILRA1*, *LILRA2*, *LILRA4*, *LILRA6*, *LILRB1*, *LILRB2*, *LILRB3*, and *LILRB4* had a statistically significant lower expression in cancer tissues (*p* < 0.05; Figure 1a). We then performed correlation analyses on these 10 LILR genes and visualised the results with correlation diagrams as shown in Figure 1b. The results showed that they were all positive correlations, and most of them were strong correlations (correlation coefficient ≥0.6). Some of them were very strong correlations (correlation coefficient ≥0.8), such as *LILRB1* and *LILRB4*, *LILRB2*, and *LILRB3*.

**FIGURE 2:**
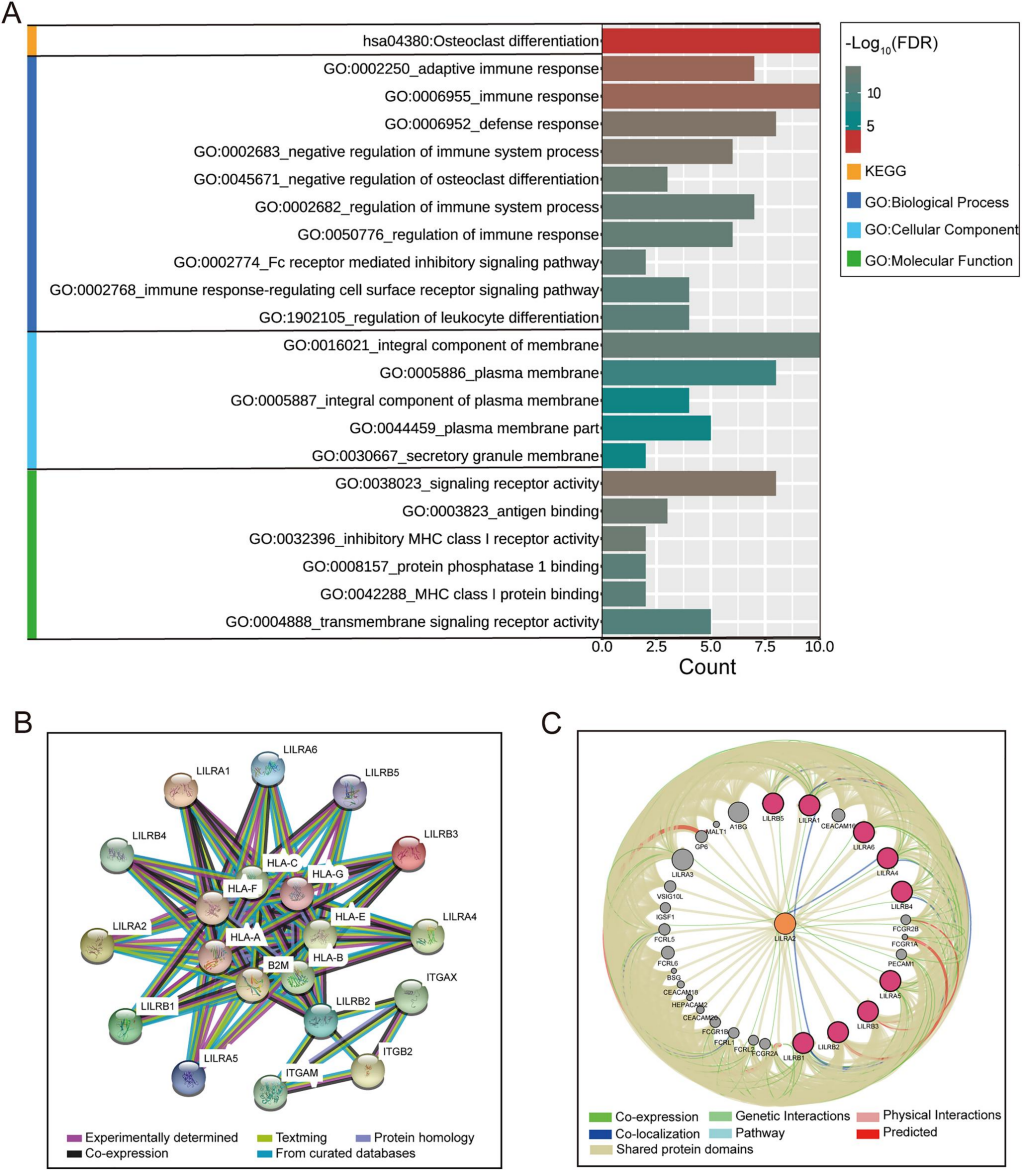
The analysis of the Kyoto Encyclopaedia of Genes and Genomes (KEGG) pathway, Gene Ontology (GO), and gene/protein interaction for LILR genes. (a) Visualisation of the KEGG pathway and GO analysis results. (b) The protein–protein interaction networks of LILR genes, which were derived from STRING. (c) The gene–gene interaction networks of LILR genes, which were derived from GeneMANIA.

Furthermore, we conducted functional annotation and pathway enrichment for the LILR genes. As shown in the results of Figure 2a and Table S2, the LILR genes were mainly involved in the adaptive immune response, the regulation of immune response, the immune system process, leucocyte differentiation, MHC class I protein binding, transmembrane signalling receptor activity, and inhibitory MHC class I receptor activity. Notably, the Fc receptor‐mediated inhibitory signalling pathway, immune response‐regulating cell surface receptor signalling pathway, regulation of immune response, regulation of immune system process, and immune response were in the same regulatory network, which suggested that the LILRs genes played an important role in the immune response and regulation. The results of protein‐protein interaction (PPI) analysis showed that LILR genes were closely associated with each other, and they were related to the HLA family and integrin family genes (Figure 2b). The results from GeneMANIA were similar, showing a close relationship between LILR genes (Figure 2c).

### 3.3 Survival analysis and prognostic model construction

In the analysis of the association between the above clinical factors and clinical outcomes, we found that four variables (neoplasm histological grade, targeted molecular therapy, radiation therapy, and residual resection) were significantly associated with OS, and two factors (neoplasm histological grade and residual resection) were significantly associated with RFS as shown in Table S1. Clinical factors of appeal were included in the Cox proportional hazard regression model as corrective factors. Other factors were not significantly associated with clinical outcomes.

**FIGURE 3:**
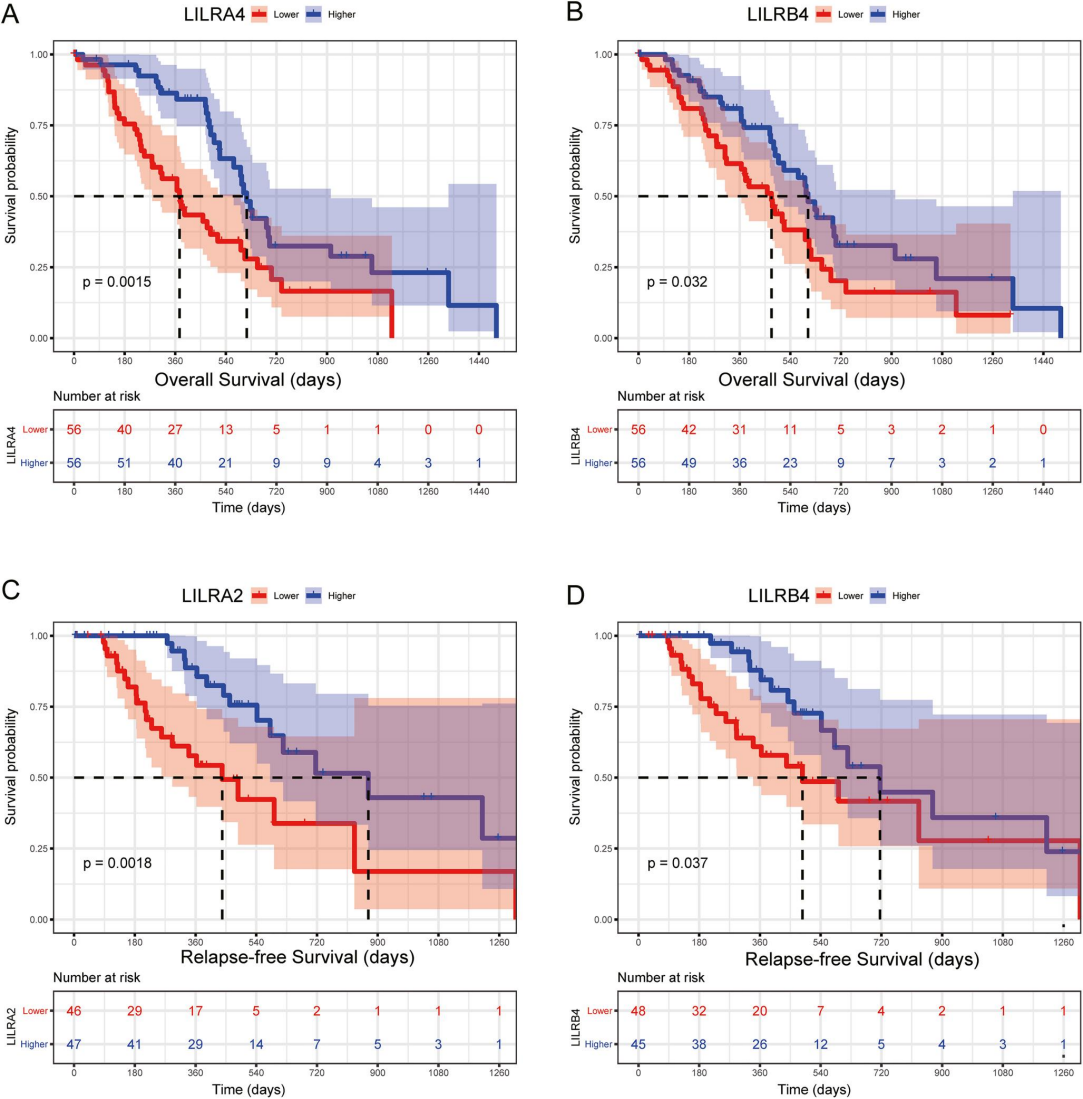
A Kaplan–Meier plot of the association between LILR gene expressions and clinical outcomes in early‐stage PDAC. OS for *LILRA4* (a) and *LILRB4* (b); RFS for *LILRA2* (c), and *LILRB4* (d).

The Kaplan–Meier analyses showed a significant association between *LILRA2*, *LILRA4*, *LILRA6*, *LILRB1*, *LILRB3*, *LILRB4*, and *LILRB5* and OS in early‐stage PDAC patients with pancreaticoduodenectomy (Figure 3; Figure S1A in Supporting Information). Multivariate analysis results of Cox proportional hazard regression models after received clinical factor adjustment showed that the high expression of *LILRA4* (*p* = 0.006, HR = 0.46, 95% CI: 0.27–0.80) and *LILRB4* (*p* = 0.04, HR = 0.57, 95% CI: 0.33–097) were still significantly associated with a better OS (Table 1 and Figure 3a,b). In a univariate analysis of RFS of Kaplan–Meier analyses, we found that *LILRA2*, *LILRA4*, and *LILRB4* were significantly associated with RFS (Figure S1B Supporting Information). In the multivariate Cox proportional hazard regression model, there were significant associations between the high expression of *LILRA2* (*p* = 0.008, HR = 0.35, 95% CI: 0.16–0.76) and *LILRB4* (*p* = 0.038, HR = 0.46, 95% CI: 0.22–0.96) with a better RFS (Table 1 and Figure 3c,d).

**TABLE 1:** Analysis of the association between LILRs gene expression and clinical outcomes.

Gene	Overall survival				Relapse‐free survival			
	Patients	MST (days)	HR (95% CI)	*p* ^#^	Patients	MST (days)	HR (95% CI)	p*
LILRA1								
Lower	56	473	Reference		46	486	Reference	
Higher	56	596	0.60 (0.35–1.03)	0.064	47	716	0.66 (0.31–1.40)	0.281
LILRA2								
Lower	56	473	Reference		46	439	Reference	
Higher	56	596	0.58 (0.33–1.02)	0.058	47	872	0.35 (0.16–0.76)	0.008
LILRA4								
Lower	56	375	Reference		46	593	Reference	
Higher	56	614	0.46 (0.27–0.80)	0.006	47	716	0.50 (0.23–1.04)	0.065
LILRA5								
Lower	56	485	Reference		48	542	Reference	
Higher	56	568	0.82 (0.49–1.38)	0.454	45	831	0.80 (0.39–1.63)	0.537
LILRA6								
Lower	56	481	Reference		45	593	Reference	
Higher	56	614	0.91 (0.52–1.60)	0.735	48	620	0.75 (0.37–1.50)	0.411
LILRB1								
Lower	56	498	Reference		45	486	Reference	
Higher	56	592	0.68 (0.40–1.17)	0.164	48	716	0.59 (0.27–1.27)	0.178
LILRB2								
Lower	56	485	Reference		46	486	Reference	
Higher	56	592	0.85 (0.50–1.45)	0.552	47	831	0.61 (0.30–1.28)	0.193
LILRB3								
Lower	56	458	Reference		46	593	Reference	
Higher	56	614	0.59 (0.34–1.04)	0.066	47	716	0.60 (0.29–1.23)	0.162
LILRB4								
Lower	56	473	Reference		48	486	Reference	
Higher	56	603	0.57 (0.33–097)	0.040	45	716	0.46 (0.22–0.96)	0.038
LILRB5								
Lower	56	476	Reference		44	593	Reference	
Higher	56	596	0.75 (0.43–1.31)	0.318	49	716	0.80 (0.38–1.66)	0.545

*Note*: Multivariate cox proportional hazards regression model was ^#^adjusted by neoplasm histological grade, targeted molecular therapy, radiation therapy, residual resection in overall survival and *adjusted by Neoplasm histological grade, residual resection in relapse‐free survival.

Abbreviations: CI, confidence interval; HR, hazard ratio; LILRs, leukocyte immunoglobulin‐like receptors; MST, median, survival time.

**FIGURE 4:**
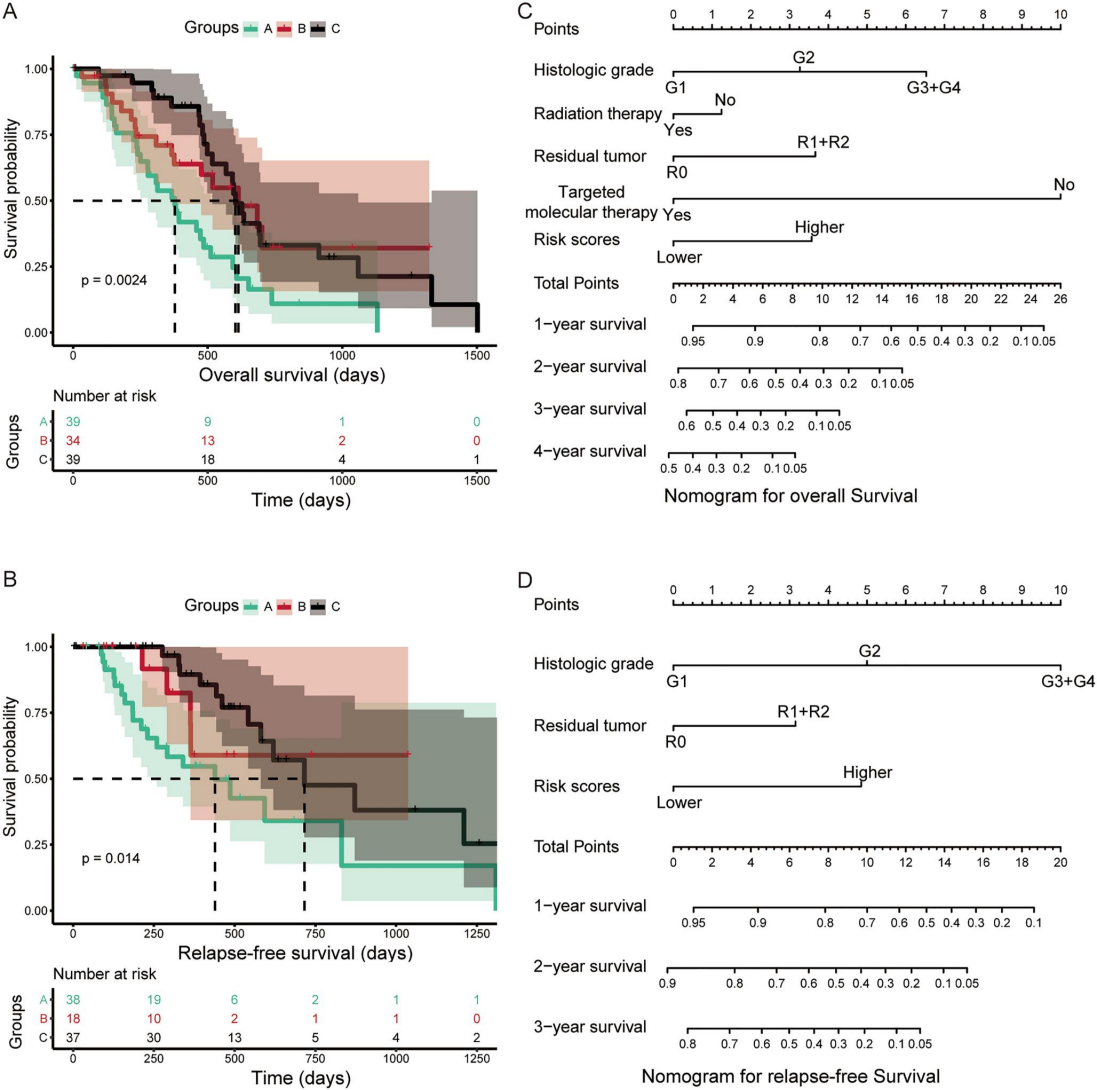
Combined survival analysis of LILRs gene expressions with prognoses for early‐stage PDAC, and a nomogram plot for early‐stage PDAC. (a) Kaplan–Meier plot for *LILRA4* and *LILRB4*, *n* = 112 (b) *LILRA2* and *LILRB4*, *n* = 93, Kaplan–Meier analysis. A nomogram plot for OS (c), RFS (d).

We then conducted a joint analysis of genes that were significantly associated with clinical outcomes based on gene expressions. According to the level of *LILRA4* and *LILRB4* expressions, we divided PDAC patients into three subgroups: group A (both *LILRA4* and *LILRB4* had low expressions), group B (a combination of high and low expressions), and group C (both *LILRA4* and *LILRB4* had high expressions). The results are shown in Table 2 and Figure 4a. Compared with group A, Group C was significantly associated with better OS (*p* = 0.007, HR = 0.42, 95% CI: 0.23–0.79). A similar result was found in the combination of *LILRA2* and *LILRB4*: there was a significant association between group C and better RFS (*p* = 0.012, HR = 0.35, 95%CI: 0.16–0.79) (Table 3 and Figure 4b).

**TABLE 2:** Combined survival analysis of LILRA4 and LILRB4 gene expression with overall survival in early‐stage PDAC.

Groups	Overall survival				
	Patients (*n* = 112)	MST	Events	HR (95% CI)	p*
LILRA4&LILRB4					
A	39	378	30	Reference	**0.023**
B	34	614	16	0.84 (0.42–1.69)	0.631
C	39	603	23	0.42 (0.23–0.79)	**0.007**

*Note*: Multivariate cox proportional hazards regression model was *adjusted by neoplasm histological grade, targeted molecular therapy, radiation therapy, and residual resection in overall survival (bold means *p*‐value < 0.05).

Abbreviations: CI, confidence interval; HR, hazard ratio; LILR, Leucocyte immunoglobulin‐like receptor; MST, median survival time.

**TABLE 3:** Combined survival analysis of LILRA2 and LILRB4 gene expressions with relapse‐free survival in early‐stage PDAC.

Groups	Relapse‐free survival				
	Patients (*n* = 93)	MST (days)	Events	HR (95% CI)	*p*
LILRA2 and LILRB4					
A	38	439	19	Reference	**0.038**
B	18	NA	4	0.54 (0.18–1.65)	0.281
C	37	716	12	0.35 (0.16–0.79)	**0.012**

*Note*: *adjusted by neoplasm histological grade and residual resection (bold means *p*‐value < 0.05).

Abbreviations: CI, confidence interval; HR, hazard ratio; MST, median survival time; PDAC, pancreatic ductal adenocarcinoma; TCGA, The Cancer Genome Atlas.

**FIGURE 5:**
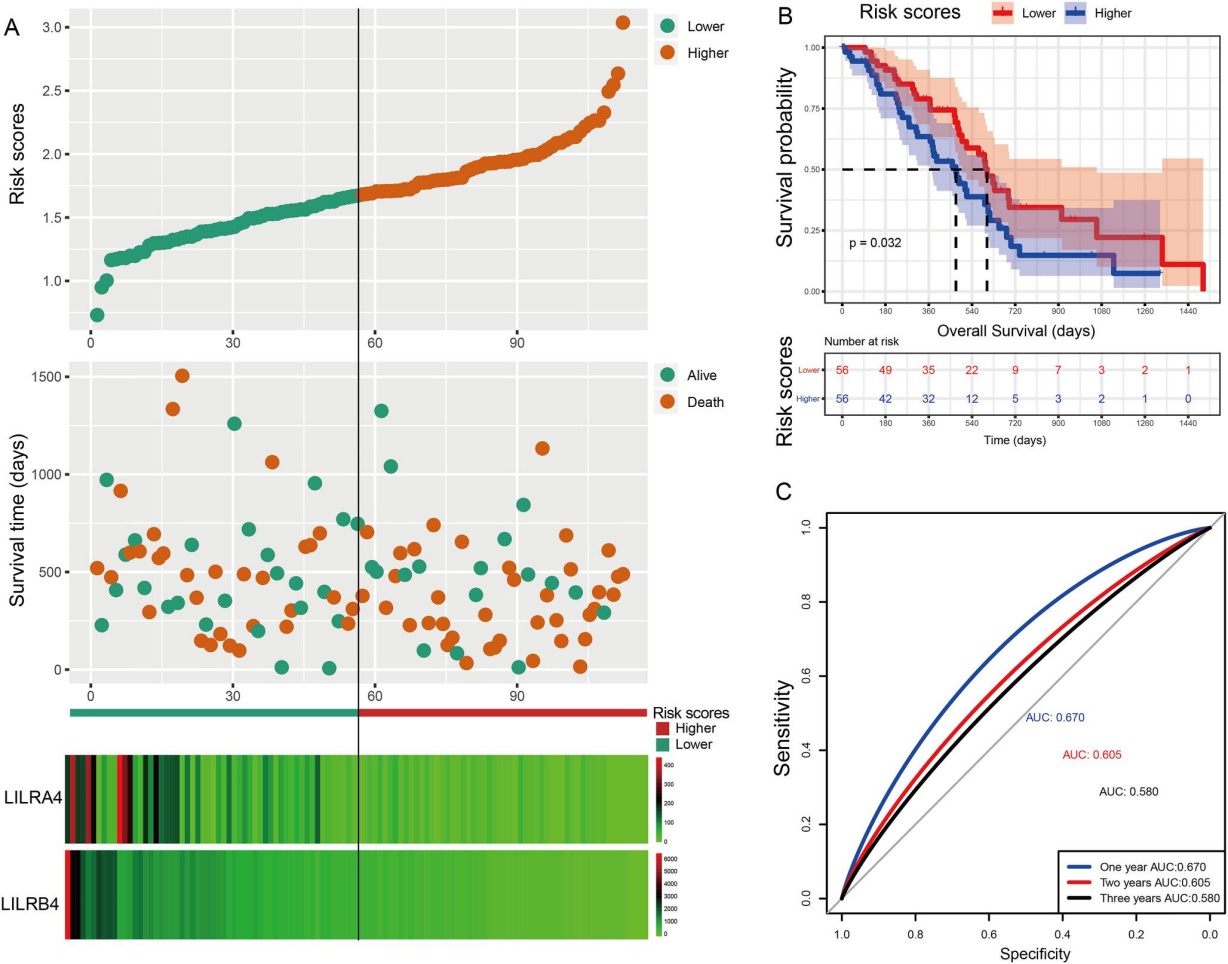
Visualisation for a prognostic model, Kaplan–Meier plot for the risk scores group, and an ROC curve for the effective power of the OS prognostic model. (a) From top to bottom: risk scores map, survival scatterplot, the heat map of the expression of *LILRA4* and *LILRB4* lower and higher groups; red represents upregulation; blue represents downregulation. (b) A Kaplan–Meier plot for the risk score group. (c) The ROC curve for the OS prognostic model.

**FIGURE 6:**
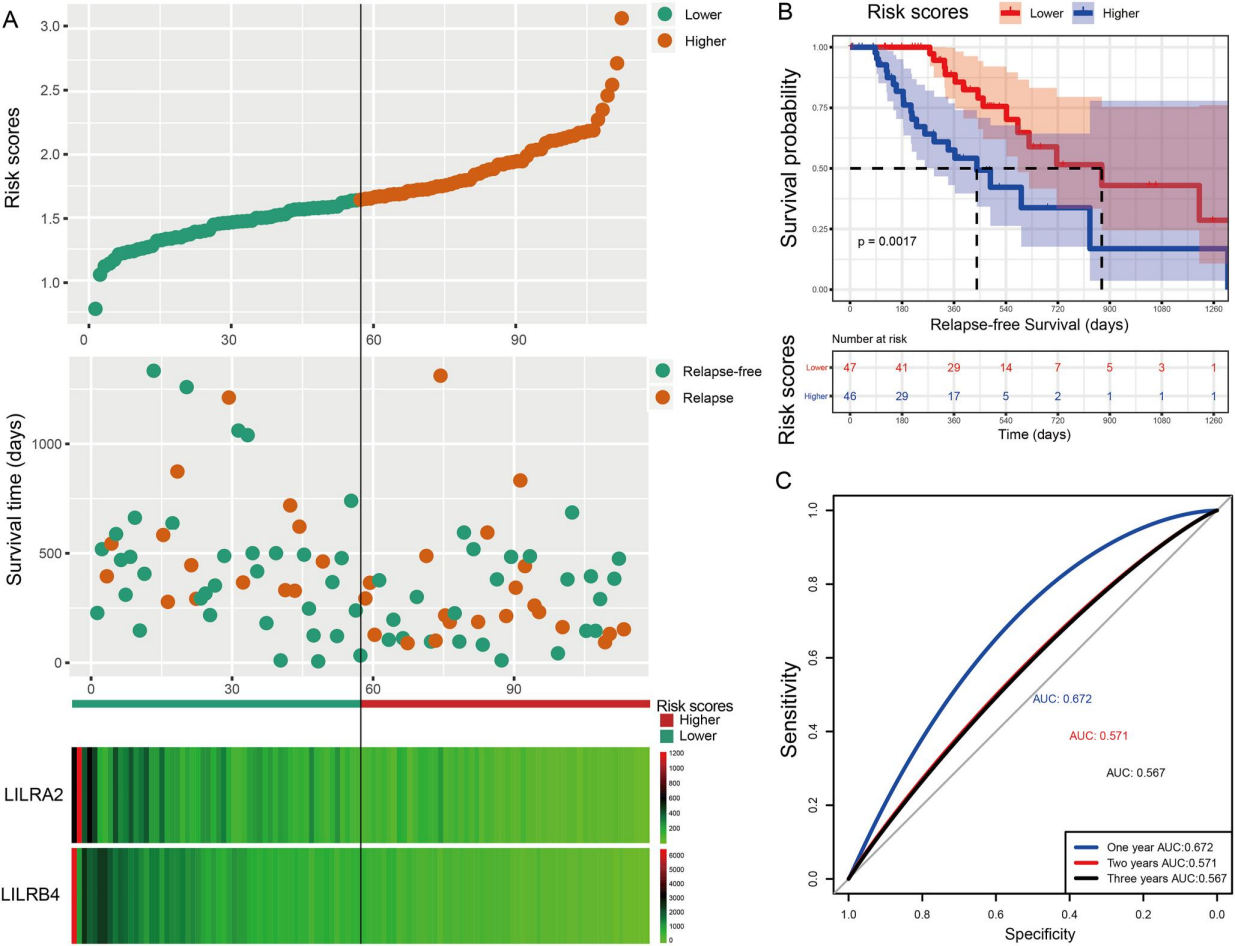
Visualisation of the prognostic model, a Kaplan–Meier plot for the risk scores group, and an ROC curve for the effective power of the RFS prognostic model. Using the median expression of each LILR gene as the cut‐off point, we defined the higher and lower risk groups for PDAC. (a) From top to bottom: risk scores map, survival scatterplot, the heat map of expression of *LILRA2* and *LILRB4* lower and higher groups; red represents upregulation; blue represents downregulation. (b) Kaplan–Meier plot for the risk score group. (c) The ROC curve for the RFS prognostic model.

Subsequently, we constructed a prognostic model for OS based on *LILRA4* and *LILRB4*, and a prognostic model for RFS was based on *LILRA2* and *LILRB4*. In this study, the β value of the Cox proportional hazard regression model was negative; so to facilitate analysis and graph display, we performed a logarithmic transformation of raw risk scores. The specific formula of the prognostic model was as follows: risk scores (OS) = ( −log10(*LILRA4* expression × −0.673 + *LILRB4* × −0.26)))+ 4, risk scores (RFS) = (‐log10‐(*LILRA2* expression × −0.123 + *LILRB4* × −0.095)])) + 4. Here, the constant 4 was used to make the final risk scores a positive number. Based on median risk scores as the cut‐off point, patients were divided into higher risk and lower risk groups. From the results of Table 4 and Figure 5a,b, we found that the higher risk score group was significantly associated with poor OS compared to the lower risk score group (*p* = 0.039, HR = 1.75, 95% CI: 1.03–3.00). In the prognostic model of RFS, the result showed that patients with PDAC after pancreaticoduodenectomy with higher risk scores suggested a poor RFS (*p* = 0.007, HR = 2.90, 95% CI: 1.33–6.15) (Table 5; Figure 6a,b). ROC curve analysis was used to evaluate the predictive effectiveness for both prognostic models. The area under the curve (AUC) of the prognostic model of OS for 1‐year, 2‐year, and 3‐year were 0.670, 0.605, and 0.580, respectively (Figure 5c), and the AUC of RFS was 0.672, 0.571, and 0.567, respectively (Figure 6c). After 5‐fold cross‐validation, the results showed that the mean AUCs of 1‐year, 2‐year and 3‐year OS predicted by the model were 0.68, 0.62, and 0.60, respectively. The mean AUCs of 1‐year, 2‐year and 3‐year RFS predicted by the model were 0.58, 0.66, and 0.62, respectively. Moreover, the nomogram results also suggested that patients with higher risk scores had a worse prognosis (Figure 4c,d) and the C‐index of OS and RFS models was 0.76 and 0.74, respectively.

**TABLE 4:** The association analysis between risk scores and overall survival in early‐stage PDAC.

Groups	Overall survival				
	Patients (*n* = 112)	MST	Events	HR (95% CI)	p*
Risk scores					
Lower	56	603	31	Reference	**0.039**
Higher	56	473	38	1.75 (1.03–3.00)	

*Note*: *adjusted by neoplasm histological grade, targeted molecular therapy, radiation therapy, and residual resection (bold means *p*‐value < 0.05).

Abbreviations: CI, confidence interval; HR, hazard ratio; MST, median survival time; PDAC, pancreatic ductal adenocarcinoma; TCGA, The Cancer Genome Atlas.

**TABLE 5:** The association analysis between risk scores and relapse‐free survival in early‐stage PDAC.

Groups	Relapse‐free survival				
	Patients (*n* = 93)	MST	Events	HR (95% CI)	p*
Risk scores					
Lower	47	14	872	Reference	0.007
Higher	46	21	493	2.90 (1.33–6.15)	

*Note*: *adjusted by neoplasm histological grade and residual resection.

Abbreviations: CI, confidence interval; HR, hazard ratio; MST, median survival time; PDAC, pancreatic ductal adenocarcinoma; TCGA, The Cancer Genome Atlas.

Analysis of the tumour biopsies revealed that LILRA2, LILRA4, and *LILRB4* were associated with the prognosis of PDAC patients. To test if these genes had prognostic potential in PBMCs as well, GSE49641 and GSE74629 datasets were analysed. We found that the difference in the expression of these genes in cancer patients and the healthy controls was not statistically significant in GSE49641 (Figure S2A‐C Supporting Information), but LILRA2 and *LILRB4* were significantly higher in the peripheral blood of cancer patients in GSE74629 (Figure S2D‐F Supporting Information). Due to significant differences in the expression of LILRA2 and LILRB4 in GSE74629 in peripheral blood of PDAC patients, we decided to construct an AUC curve to evaluate the diagnostic efficacy of *LILRA2* and *LILRB4* in the diagnosis of PDAC. The AUCs of *LILRA2* and *LILRB4* were 0.853 and 0.896, respectively (Figure S2G in Supporting Information), which showed the strong ability of *LILRA2* and *LILRB4* in diagnosing PDAC by detecting the expression levels in peripheral blood. Then, we set to confirm whether the lower expression of these genes is in the tumour tissue compared to the normal peri‐tumoral pancreatic tissues holds in a validation dataset, namely GSE55643. *LILRA2*, *LILRA4*, and *LILRB4* showed low expressions in tumour tissues, in which *LILRB4* was significantly lowly expressed (*p* < 0.001; Figure S3A in Supporting Information).

### 3.4 GSEA

**FIGURE 7:**
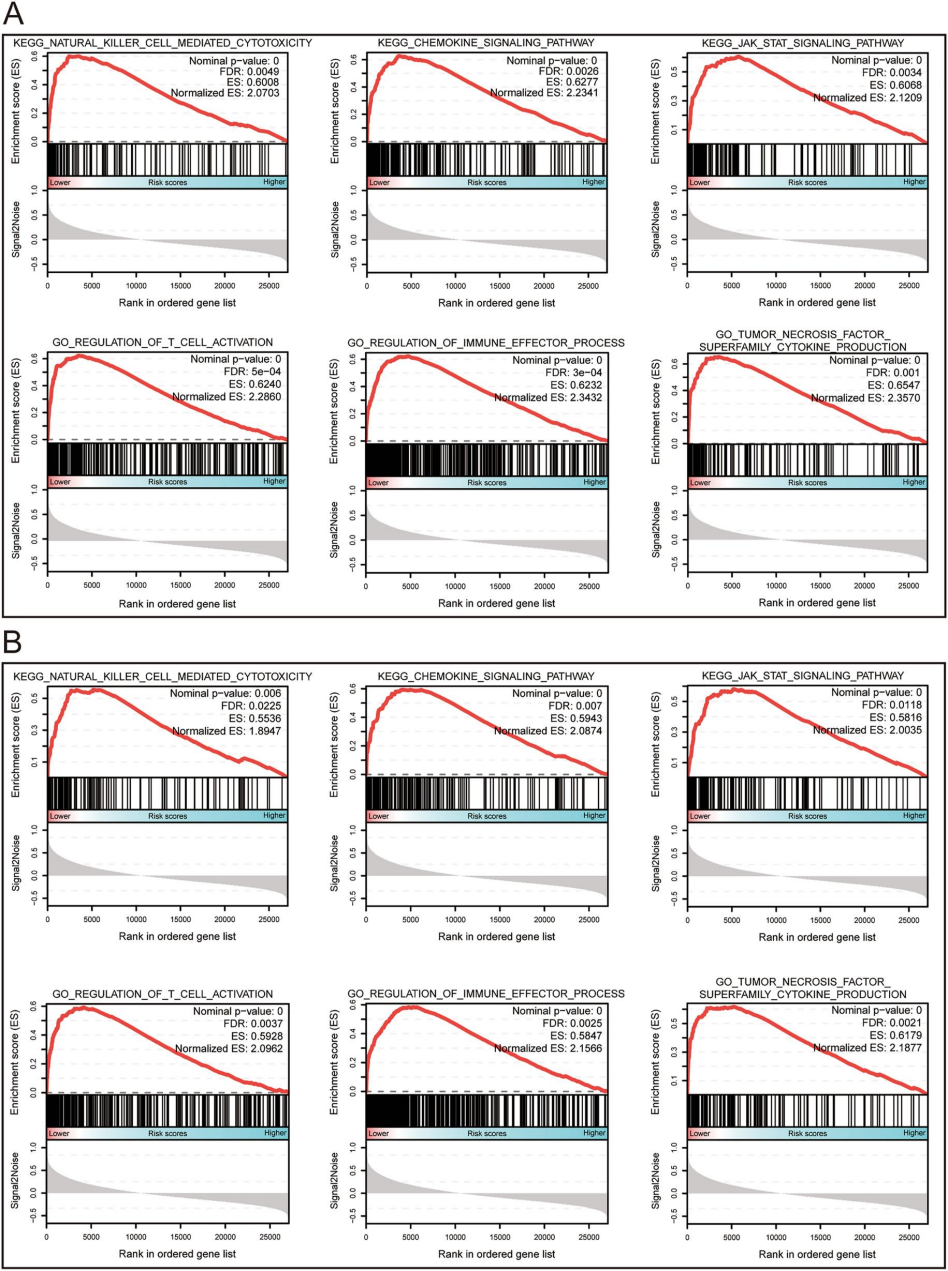
The results of GSEA for the OS (a) and RFS (b) prognostic model. The median expression of each LILR gene was used as the cut‐off point, which defined the higher and lower risk groups for the early‐stage PDAC.

GSEA was used to determine the potential molecular mechanisms that affected poor prognoses in the higher risk score groups. Our results indicated that has04650 (natural killer cell‐mediated cytotoxicity), hsa04062 (chemokine signalling), and hsa04630 (JAK‐STAT signalling pathway) were enriched in the C2 gene set, which may be involved in affecting OS and RFS (Figure 7a). In the C5 gene set, GO 0050863 (regulation of T cell activation), GO 0002697 (regulation of immune effector process), and GO 0071706 (tumour necrosis factor superfamily cytokine production) were enriched and significantly correlated with OS and RFS (Figure 7b).

### 3.5 Analysis of TIICs

**FIGURE 8:**
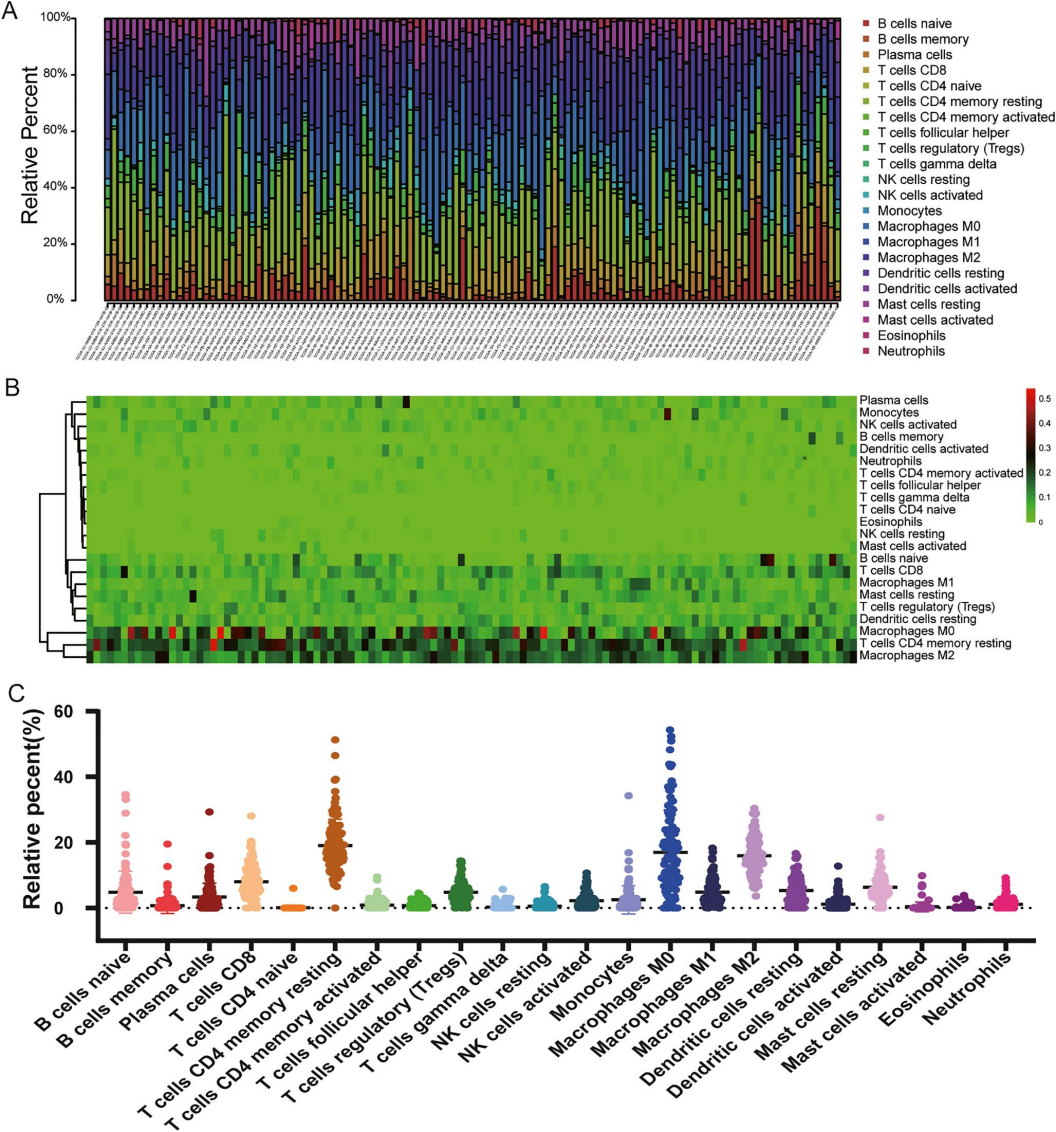
The landscape of tumour‐infiltrating immune cells for 112 early‐stage PDAC cases. (a) A histogram for infraction of 22 types of tumour‐infiltrating immune cells in each case. (b) A heat map for the fraction of 22 types of tumour‐infiltrating immune cells in each case; red represents upregulation; blue represents downregulation. (c) Tumour‐infiltrating immune cell populations for each immune cell type.

**FIGURE 9:**
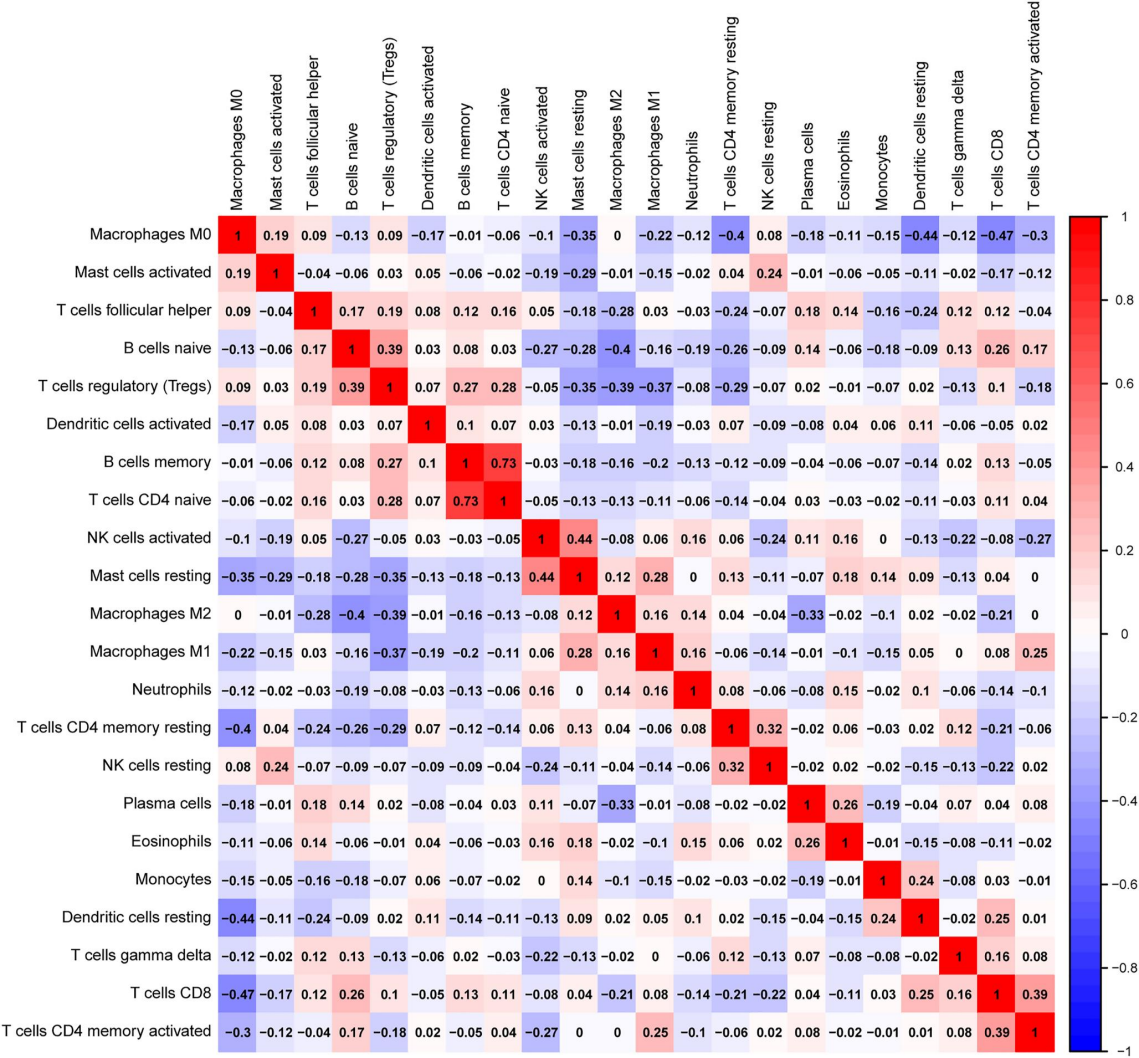
Correlogram of Pearson's correlation analysis of tumour‐infiltrating immune cells; red denotes a positive correlation, blue denotes a negative correlation, and the shade of colour represents the size of the correlation coefficient.

**FIGURE 10:**
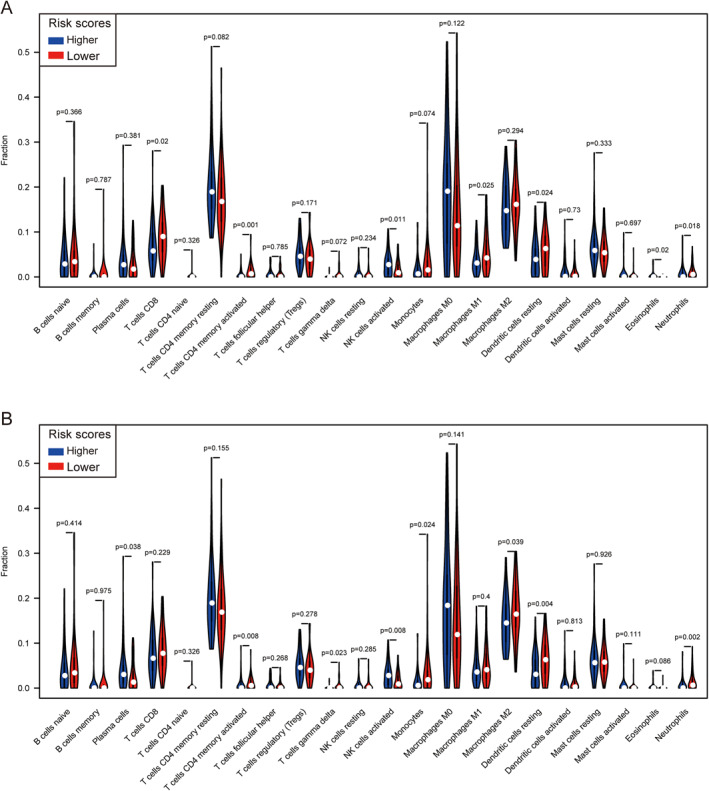
A violin diagram of differential infiltrating infraction analysis for the OS prognostic model (a) and RFS prognostic model (b).

According to the previous analysis from the TCGA database, *LILR*s may influence postoperative clinical outcomes of patients with PDAC by participating in the regulation of the immune response and the function of immune cells, but whether the immune infiltration contributed to it was unknown. Hence, we used CIBERSORT to evaluate the differential percentages of TIICs between the higher and lower risk score groups. CIBERSORT is a deconvolution algorithm based on gene expression; it is combined with 22 leucocyte gene signature matrices, which are a defined ‘barcode’ with 547 gene expression signatures to distinguish the subgroups of 22 type immune cells. Figure 8a,b shows the landscape of tumour‐infiltrating immune cells for 112 early‐stage PDAC cases. Tumour‐infiltrating immune cell populations for each immune cell type are shown in Figure 8c. We then performed correlation analyses on the relative percentages of these 22 immune cells. The result showed that there was a strong correlation between naïve CD4 T cells and memory B cells (correlation coefficient = 0.73) (Figure 9). Furthermore, the results of differential percentages of the tumour‐infiltrating immune cells showed that there was a difference in T cell CD8 (*p* = 0.02), memory‐activated CD4 T cells (*p* = 0.001), activated NK cells (*p* = 0.011), M1 macrophages (*p* = 0.025), resting dendritic cells (*p* = 0.024), eosinophils (*p* = 0.02), and neutrophils (*p* = 0.018) between both groups using the OS prognostic model (Figure 10a). In addition, the percentages of tumour‐infiltrating cells in the RFS prognostic model, including plasma cells (*p* = 0.038), memory‐activated CD4 T cells (*p* = 0.008), gamma delta T cells (*p* = 0.023), activated NK cells (*p* = 0.008), M2 macrophages (*p* = 0.039), resting dendritic cells (*p* = 0.004), and neutrophils (*p* = 0.002), were statistically significant between the two groups (Figure 10b). In addition, in order to understand the TIICs in PMBCs and solid tumour tissues, we analysed GSE74629 and found that the results were inconsistent with the analysis results in TCGA, possibly because the cell types of the components were different (Figure S3D in Supporting Information).

## 4 DISCUSSION

In this study, we investigated the association between LILR genes and clinical prognosis and diagnosis in early‐stage PDAC. Our results suggested that compared with adjacent tissues, *LILRA1*, *LILRA2*, *LILRA4*, *LILRA6*, *LILRB1*, *LILRB2*, *LILRB3*, and *LILRB4* were significantly overexpressed in cancer tissues. Multivariate analyses of the Cox proportional hazard regression model showed that higher expressions of *LILRA4* and *LILRB4* were significantly associated with better OS, and there was a significant association between lower expression of *LILRA2* and *LILRB4* and a worse RFS. However, inconsistent with the results of other tumours, high expressions of *LILRB2* and *LILRB4* were associated with better clinical outcomes in our study, while other studies showed that patients with high expressions of *LILRB2* and *LILRB4* predicted worse clinical outcomes [45, 46, 47, 48, 49, 50, 51, 52]. The inconsistency of the results was confusing and interesting for further study. Tumours are involved in an abnormally complex process, and the entire process cannot be explained by individual gene events. Therefore, we conducted a joint analysis and constructed a prognostic model based on gene expressions significantly associated with clinical outcomes. The joint effect of clinical variables and LILR expression indicated that patients with two risk factors had higher hazard ratios than those with only one risk factor. Our findings suggested that the risk score can be used to evaluate the clinical outcomes of patients with early‐stage PDAC. The AUC on the ROC curves was slightly smaller than some well‐known prognostic scores, such as the Glasgow prognostic score, or the modified Glasgow prognostic score. However, the HR value was similar [59, 60]. The difference was that the prognostic score of this study was also effective in the evaluation of RFS. Moreover, based on the prognostic model, tumour‐infiltrating immune cells were investigated. Our findings can therefore help assess the RFS for PDAC patients and identify immunotherapeutic targets.

The prognostic model included LILRA and LILRB genes, consistent with real‐world studies, and were grouped according to the level of risk score to investigate the possible mechanism of risk score influencing clinical prognosis using GSEA analysis, which may make the results more reliable. GSEA results revealed that the following pathways may be involved in regulating the potential molecular mechanisms that affect clinical prognosis; the JAK‐STAT signalling pathway, regulation of T cell activation, regulation of immune effector processes, and tumour necrosis factor superfamily cytokine production were enriched and significantly correlated with the OS and RFS. Numerous studies have shown that the activation of the JAK‐STAT signalling pathway promotes the development and progression of tumours, including pancreatic cancer [61, 62, 63, 64]. Therefore, we have a reason to speculate that in the low‐risk group, activation of the JAK‐STAT signalling pathway played an important role in affecting the OS and RFS. At the same time, many studies have reported that the JAK‐STAT signalling pathway is closely related to immune evasion, immune regulation, immune cell differentiation, and drug resistance [64, 65, 66].

The results of GSEA also showed enrichment of the immune‐related signalling pathway, which suggested that the JAK‐STAT signalling pathway seems to have some association. Hence, we investigated the differential percentages of TIICs between the higher and lower risk score groups. Our results found that in a prognostic model of OS and RFS, there were significantly different percentages in memory‐activated CD4 T cells, activated NK cells, resting dendritic cells, and neutrophils between the two groups. A series of studies have shown that TIICs are closely related to tumour development, drug response, and clinical prognoses in a variety of tumours [67, 68, 69, 70, 71, 72]. In these studies, a high fraction of infiltrating and activated CD4 memory T cells, resting dendritic cells, and neutrophils indicated a good clinical prognosis, which is consistent with previous studies. However, in contrast to previous studies, the high‐risk group had high percentages of activated NK cells, while the low‐risk group had lower fractions of activated NK cells, which was similar to the relationship between the expression of *LILRB4* and clinical prognosis in early‐stage PDAC. In general, NK cells play an anti‐tumour role and are regarded as prognostic factors for a good prognosis in some tumours, but tumour‐infiltrating NK cells may also play a role in promoting tumours [73]. NK cells are divided into two subtypes according to the cell surface antigens CD56 and CD16; namely CD56dim/CD16+ and CD56bright/CD16‐. CD56dim/CD16+ NK cells have a high cytotoxic potential, while the CD56bright/CD16‐subtype has a low cytotoxic potential but can secrete cytokines that promote angiogenesis. The vast majority of tumour‐infiltrating NK cells are the CD56bright/CD16‐subtype and have been reported to have impaired cytotoxicity and cytokine‐producing functions and promote angiogenesis, thus playing a role in promoting tumours [74, 75]. Therefore, it is possible that in the high‐risk group, a high fraction of tumour‐infiltrating NK cells plays a role in promoting tumours, which is a reasonable explanation. The infiltrating immune cells are part of the tumour microenvironment. In the prognosis model, the combined effect of the infiltrating immune cells indicates the possible mechanism of poor prognosis in the high‐risk group. The bioinformatics analysis in solid tissues was based on a complex statistical method and was calculated using a formula. CIBERSORT was verified in lung specimens obtained during surgical resection of early‐stage non‐small‐cell lung carcinomas and disaggregated lymph node biopsies from follicular lymphoma by flow cytometry. The results of CIBERSORT were significantly correlated with flow cytometry measurements ([76]), but the real correlation in PDAC needs to be verified by additional studies.

## 5 CONCLUSION

In this study, we investigated the associations between LILRs genes and clinical prognosis in early‐stage PDAC. The results revealed that *LILRA4* and *LILRB4* were significantly associated with OS, and *LILRA2* and *LILRB4* were associated with the RFS. *LILRB4* was significantly related to the prognosis of PDAC patients, but the prognostic values of *LILRA2* and *LILRA4* need further validation. The prognostic model suggested that patients with early‐stage PDAC with higher risk scores had worse clinical outcomes. The results of potential molecular mechanistic analyses suggested that in the prognostic model, the JAK‐STAT signalling pathway, immune‐related signalling pathways, and tumour‐infiltrating immune cells may play a crucial role in affecting the clinical outcomes, but further functional experiments are needed for confirmation.

## AUTHOR CONTRIBUTION

**Qiang Gao**: Conceptualisation, Data curation, Writing – original draft; **Shutian Mo**: Formal analysis, Methodology; **Chuangye Han**: Formal analysis, Methodology; **Xiwen Liao**: Formal analysis, Methodology; **Chengkun Yang**: Formal analysis, Resources; **Xiangkun Wang**: Formal analysis; **Tianyi Liang**: Formal analysis; **Yongfei He**: Formal analysis; **Zijun Chen**: Formal analysis; **Guangzhi Zhu**: Formal analysis; **Hao Su**: Formal analysis; **Xinping Ye**: Formal analysis; **Tao Peng**: Conceptualisation, Funding acquisition, Project administration; Writing – review and editing.

## CONFLICT OF INTEREST STATEMENT

No potential conflict of interest was reported by the authors.

## Supporting information

Supporting Information S1

Table S1

Table S2

Table S3

## Data Availability

The raw data supporting the conclusion of this article will be made available by the authors, without undue reservation.
