# Supplementary material for: Comprehensive analysis of LILR family genes expression and tumour‐infiltrating immune cells in early‐stage pancreatic ductal adenocarcinoma
Source: IET Syst Biol. 2023 Feb 7;17(2):39–57. doi: 10.1049/syb2.12058 (PMC10116025; doi:10.1049/syb2.12058)
Supplement: Supplementary file 1 — Supporting Information S1 [file SYB2-17-39-s001.docx]

**Figure Legends**

**
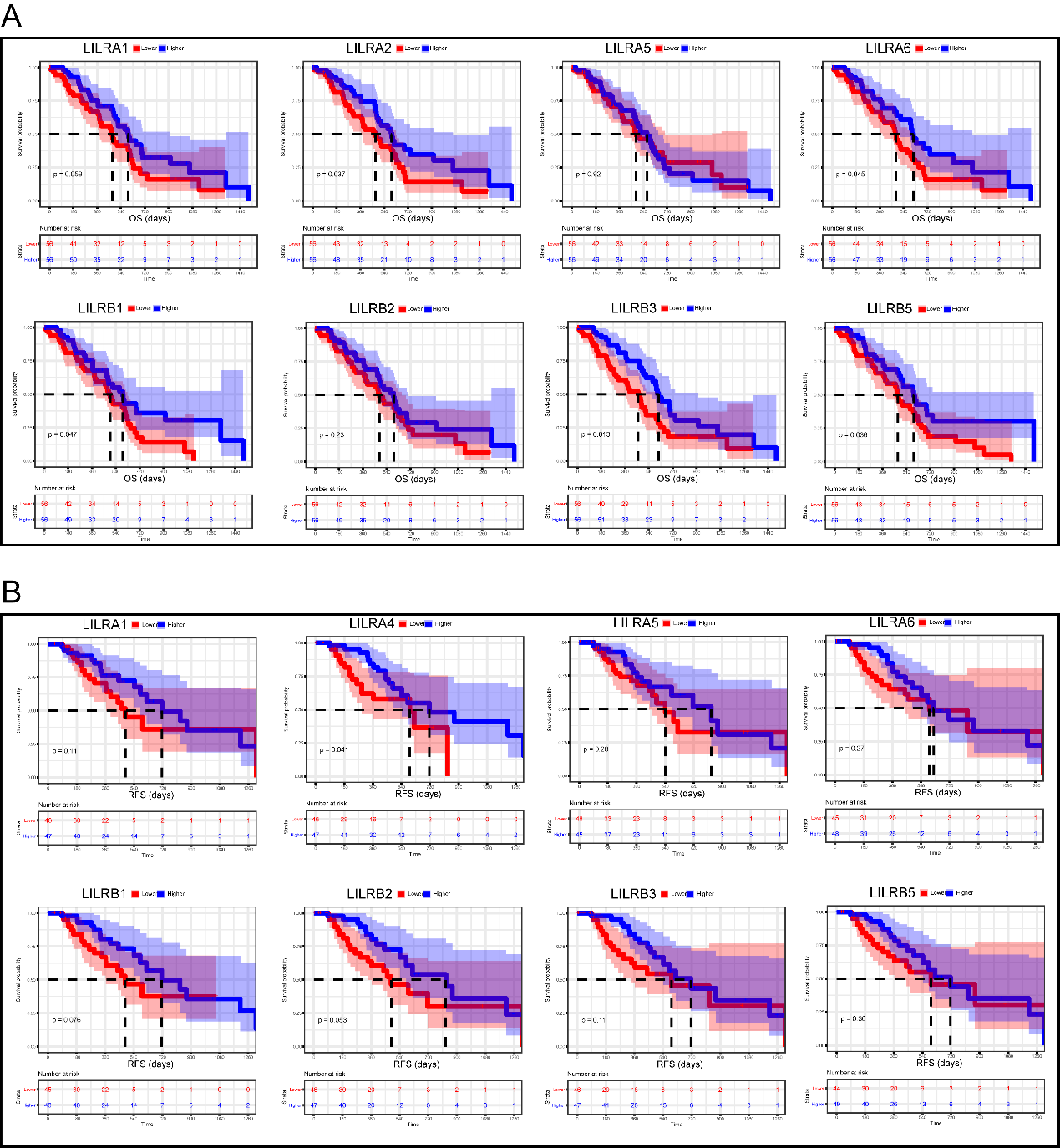
**

**Figure S1.** Kaplan–Meier plot of the association between LILR gene expressions and clinical outcomes in early-stage pancreatic ductal adenocarcinomas. Overall survival for LILRs (A) and the relapse-free survival (B).


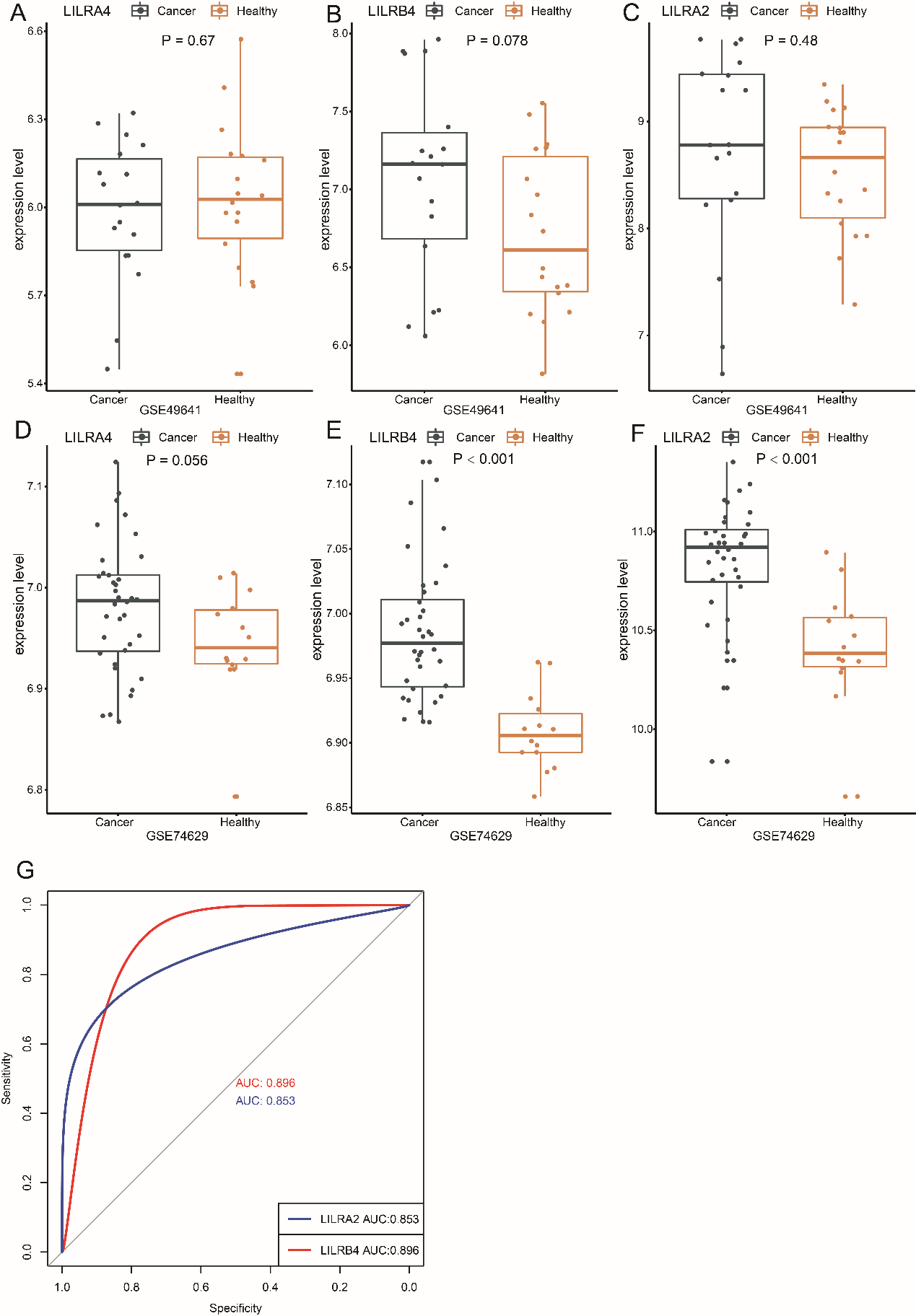


**Figure S2.** The expression analysis of *LILRA2* (A), *LILRA4* (B), and *LILRB4* (C) in GSE49641 and *LILRA2* (D), *LILRA4* (E), and *LILRB4* (F) in GSE74629. (G) The ability of *LILRA2* and *LILRB4* in diagnosing pancreatic ductal adenocarcinomas by detecting the expression levels in peripheral blood of GSE74629.

**
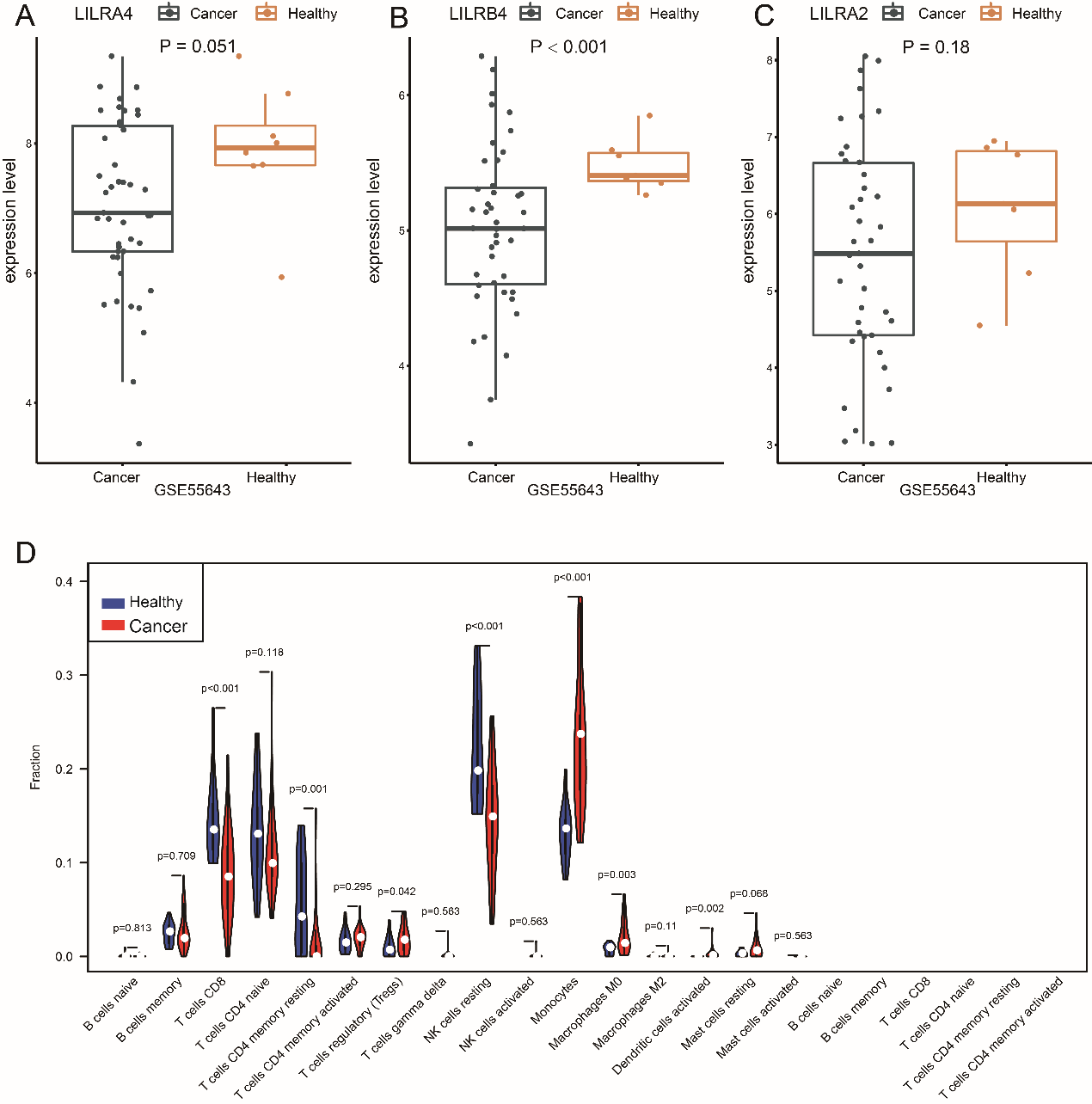
**

**Figure S3.** The expression analyses of *LILRA2* (A), *LILRA4* (B), and *LILRB4* (C) in GSE55643. (D) A violin diagram of differential tumor-infiltrating infraction analysis in GSE74629.
