# Supplementary material for: Comprehensive analysis of LILR family genes expression and tumour‐infiltrating immune cells in early‐stage pancreatic ductal adenocarcinoma
Source: IET Syst Biol. 2023 Feb 7;17(2):39–57. doi: 10.1049/syb2.12058 (PMC10116025; doi:10.1049/syb2.12058)
Supplement: Supplementary file 2 — Table S1 [file SYB2-17-39-s003.docx]

**Table S1**. Clinicopathological characteristics and clinical prognosis of 112 early-stage PDAC, data from TCGA.

| Variable | Overall survival | | | | Relapse-free survival | | | |
| --- | --- | --- | --- | --- | --- | --- | --- | --- |
|  | Patients (n=112) | MTS (days) | HR (95% CI) | *P* | Patients(n=93) | MST (days) | HR (95% CI) | *P* |
| Gender |  |  |  |  |  |  |  |  |
| Male | 59 | 592 | Refrence |  | 50 | 593 | Refrence |  |
| Female | 53 | 511 | 1.17 (0.72—1.89) | 0.523 | 43 | 831 | 0.99 (0.51—1.93) | 0.971 |
| Age(years) |  |  |  |  |  |  |  |  |
| ≤60 | 38 | 593 | Refrence |  | 31 | 716 | Refrence |  |
| >60 | 74 | 485 | 1.64 (0.96—2.78) | 0.069 | 62 | 581 | 0.81 (0.42—1.60) | 0.55 |
| Alcohol history |  |  |  |  |  |  |  |  |
| NO | 43 | 592 | Refrence |  | 38 | 716 | Refrence |  |
| YES | 61 | 511 | 1.28 (0.77—2.13) | 0.350 | 48 | 593 | 1.29 (0.63—2.64) | 0.480 |
| NA | 8 |  |  |  | 5 |  |  |  |
| History of chronic pancreatitis |  |  |  |  |  |  |  |  |
| NO | 79 | 511 | Refrence |  | 62 | 581 | Refrence |  |
| YES | 9 | 607 | 1.34 (0.60—2.97) | 0.479 | 8 | NA | 0.26 (0.036—1.95) | 0.192 |
| NA | 24 |  |  |  | 23 |  |  |  |
| Tumor size |  |  |  |  |  |  |  |  |
| < 4cm | 66 | 517 | Refrence |  | 56 | 593 | Refrence |  |
| ≥ 4cm | 44 | 498 | 0.90 (0.56—1.45) | 0.655 | 36 | 542 | 1.22 (0.61—2.42) | 0.573 |
| NA | 2 |  |  |  | 1 |  |  |  |
| Pathological stage | |  |  |  |  |  |  |  |
| I | 8 | 518 | Refrence |  | 7 | NA | Refrence |  |
| II | 104 | 236 | 1.04 (0.38—2.87) | 0.943 | 86 | 620 | 2.34 (0.33—17.60) | 0.390 |
| Neoplasm histological grade | |  |  |  |  |  |  |  |
| G1 | 15 | 518 | Refrence |  | 12 | 620 | Refrence |  |
| G2 | 65 | 603 | 1.22 (0.54—2.76) |  | 54 | 831 | 0.74 (0.27—2.03) |  |
| G3+G4 | 32 | 470 | 2.27 (0.96—5.34) | 0.038 | 27 | 393 | 2.74 (0.94—8.02) | **0.030** |
| Targeted molecular therapy | |  |  |  |  |  |  |  |
| NO | 29 | 224 | Refrence |  | 25 | NA | Refrence |  |
| YES | 73 | 634 | 0.17 (0.095—0.30) | <0.001 | 62 | 593 | 0.72 (2.90—1.78) | 0.472 |
| NA | 10 |  |  |  | 5 |  |  |  |
| Radiation therapy |  |  |  |  |  |  |  |  |
| NO | 70 | 473 | Refrence |  | 61 | 716 | Refrence |  |
| YES | 30 | 691 | 0.53 (0.29—0.95) | 0.032 | 24 | 542 | 1.15 (0.56—2.36) | 0.698 |
| NA | 10 |  |  |  | 8 |  |  |  |
| Residual resection | |  |  |  |  |  |  |  |
| NO | 66 | 603 | Refrence |  | 51 | 831 | Refrence |  |
| YES | 44 | 381 | 1.95 (1.17—3.22) | 0.01 | 40 | 461 | 2.49 (1.17—5.28) | **0.018** |
| NA | 2 |  |  |  | 2 |  |  |  |

**Abbreviations**: PDAC, pancreatic ductal adenocarcinoma; TCGA, The Cancer Genome Atlas; HR, hazard ratio; CI, confidence interval；NA, Not Available; MST, median survival time.
