# Supplementary material for: Comprehensive analysis of LILR family genes expression and tumour‐infiltrating immune cells in early‐stage pancreatic ductal adenocarcinoma
Source: IET Syst Biol. 2023 Feb 7;17(2):39–57. doi: 10.1049/syb2.12058 (PMC10116025; doi:10.1049/syb2.12058)
Supplement: Supplementary file 3 — Table S2 [file SYB2-17-39-s004.docx]

**Table S2**. The results of KEGG pathway and Gene Ontology.

| Category | Term ID | Description | Counts | | FDR | Genes |
| --- | --- | --- | --- | --- | --- | --- |
| KEGG | hsa04380 | Osteoclast differentiation | | 10 | 3.21E-22 | LILRA1,LILRA2,LILRA4,LILRA5,LILRA6,LILRB1,LILRB2,LILRB3,LILRB4,LILRB5 |
| GO_BP | GO:0002250 | adaptive immune response | | 7 | 4.96E-09 | LILRA1,LILRA6,LILRB1,LILRB2,LILRB3,LILRB4,LILRB5 |
| GO_BP | GO:0006955 | immune response | | 10 | 4.96E-09 | LILRA1,LILRA2,LILRA4,LILRA5,LILRA6,LILRB1,LILRB2,LILRB3,LILRB4,LILRB5 |
| GO_BP | GO:0006952 | defense response | | 8 | 1.19E-06 | LILRA1,LILRA2,LILRA4,LILRA5,LILRB1,LILRB2,LILRB3,LILRB5 |
| GO_BP | GO:0002683 | negative regulation of immune system process | | 6 | 1.99E-06 | LILRA2,LILRA4,LILRB1,LILRB2,LILRB3,LILRB4 |
| GO_BP | GO:0045671 | negative regulation of osteoclast differentiation | | 3 | 3.30E-05 | LILRB1,LILRB3,LILRB4 |
| GO_BP | GO:0002682 | regulation of immune system process | | 7 | 6.12E-05 | LILRA1,LILRA2,LILRA4,LILRB1,LILRB2,LILRB3,LILRB4 |
| GO_BP | GO:0050776 | regulation of immune response | | 6 | 8.39E-05 | LILRA1,LILRA2,LILRA4,LILRB1,LILRB2,LILRB4 |
| GO_BP | GO:0002774 | Fc receptor mediated inhibitory signaling pathway | | 2 | 0.00011 | LILRB1,LILRB2 |
| GO_BP | GO:0002768 | immune response-regulating cell surface receptor signaling pathway | | 4 | 0.00025 | LILRA2,LILRA4,LILRB1,LILRB2 |
| GO_BP | GO:1902105 | regulation of leukocyte differentiation | | 4 | 0.00025 | LILRB1,LILRB2,LILRB3,LILRB4 |
| GO_BP | GO:2001198 | regulation of dendritic cell differentiation | | 2 | 0.00047 | LILRB1,LILRB2 |
| GO_BP | GO:0007166 | cell surface receptor signaling pathway | | 7 | 0.00048 | LILRA1,LILRA2,LILRA4,LILRB1,LILRB2,LILRB3,LILRB5 |
| GO_BP | GO:0045591 | positive regulation of regulatory T cell differentiation | | 2 | 0.00048 | LILRB2,LILRB4 |
| GO_BP | GO:0050870 | positive regulation of T cell activation | | 3 | 0.0019 | LILRB1,LILRB2,LILRB4 |
| GO_BP | GO:0002366 | leukocyte activation involved in immune response | | 4 | 0.0028 | LILRA2,LILRB1,LILRB2,LILRB3 |
| GO_BP | GO:0034122 | negative regulation of toll-like receptor signaling pathway | | 2 | 0.0028 | LILRA2,LILRA4 |
| GO_BP | GO:0032480 | negative regulation of type I interferon production | | 2 | 0.0031 | LILRA4,LILRB1 |
| GO_BP | GO:0032720 | negative regulation of tumor necrosis factor production | | 2 | 0.0036 | LILRA4,LILRB1 |
| GO_BP | GO:0007165 | signal transduction | | 8 | 0.0039 | LILRA1,LILRA2,LILRA4,LILRB1,LILRB2,LILRB3,LILRB4,LILRB5 |
| GO_BP | GO:0042130 | negative regulation of T cell proliferation | | 2 | 0.0045 | LILRB1,LILRB2 |
| GO_BP | GO:0051926 | negative regulation of calcium ion transport | | 2 | 0.0051 | LILRB1,LILRB2 |
| GO_BP | GO:0002684 | positive regulation of immune system process | | 4 | 0.0063 | LILRA2,LILRB1,LILRB2,LILRB4 |
| GO_BP | GO:0045088 | regulation of innate immune response | | 3 | 0.0063 | LILRA2,LILRA4,LILRB1 |
| GO_BP | GO:0051241 | negative regulation of multicellular organismal process | | 4 | 0.011 | LILRA4,LILRB1,LILRB3,LILRB4 |
| GO_BP | GO:0002283 | neutrophil activation involved in immune response | | 3 | 0.0112 | LILRA2,LILRB2,LILRB3 |
| GO_BP | GO:0002831 | regulation of response to biotic stimulus | | 2 | 0.0124 | LILRA2,LILRB1 |
| GO_BP | GO:0001816 | cytokine production | | 2 | 0.013 | LILRA2,LILRB1 |
| GO_BP | GO:0071222 | cellular response to lipopolysaccharide | | 2 | 0.014 | LILRB1,LILRB2 |
| GO_BP | GO:0001817 | regulation of cytokine production | | 3 | 0.0167 | LILRA4,LILRB1,LILRB2 |
| GO_BP | GO:0045087 | innate immune response | | 3 | 0.0207 | LILRA2,LILRA4,LILRA5 |
| GO_BP | GO:0048585 | negative regulation of response to stimulus | | 4 | 0.0228 | LILRA2,LILRA4,LILRB1,LILRB4 |
| GO_BP | GO:0051239 | regulation of multicellular organismal process | | 5 | 0.035 | LILRA4,LILRB1,LILRB2,LILRB3,LILRB4 |
| GO_BP | GO:0032940 | secretion by cell | | 3 | 0.0468 | LILRB1,LILRB2,LILRB3 |
| GO_MF | GO:0038023 | signaling receptor activity | | 8 | 9.45E-07 | LILRA1,LILRA2,LILRA4,LILRB1,LILRB2,LILRB3,LILRB4,LILRB5 |
| GO_MF | GO:0003823 | antigen binding | | 3 | 2.27E-05 | LILRA1,LILRA2,LILRB4 |
| GO_MF | GO:0032396 | inhibitory MHC class I receptor activity | | 2 | 2.27E-05 | LILRB1,LILRB2 |
| GO_MF | GO:0008157 | protein phosphatase 1 binding | | 2 | 0.00024 | LILRB1,LILRB2 |
| GO_MF | GO:0042288 | MHC class I protein binding | | 2 | 0.00024 | LILRB1,LILRB2 |
| GO_MF | GO:0004888 | transmembrane signaling receptor activity | | 5 | 0.00061 | LILRA1,LILRB1,LILRB2,LILRB3,LILRB5 |
| GO_CC | GO:0016021 | integral component of membrane | | 10 | 7.80E-05 | LILRA1,LILRA2,LILRA4,LILRA5,LILRA6,LILRB1,LILRB2,LILRB3,LILRB4,LILRB5 |
| GO_CC | GO:0005886 | plasma membrane | | 8 | 0.0059 | LILRA1,LILRA2,LILRA4,LILRA5,LILRB1,LILRB2,LILRB3,LILRB4 |
| GO_CC | GO:0005887 | integral component of plasma membrane | | 4 | 0.0399 | LILRA2,LILRA4,LILRB2,LILRB3 |
| GO_CC | GO:0044459 | plasma membrane part | | 5 | 0.0399 | LILRA2,LILRA4,LILRB1,LILRB2,LILRB3 |
| GO_CC | GO:0030667 | secretory granule membrane | | 2 | 0.0466 | LILRB2,LILRB3 |

Abbreviations: KEGG, Kyoto Encyclopedia of Genes and Genomes; FDR, False Discovery Rates; GO, Gene Ontology, MF, Molecular Function; BP, Biological Process;

CC, Cellular Component, LILR, leukocyte immunoglobulin-like receptor.
