# Supplementary material for: Comprehensive analysis of LILR family genes expression and tumour‐infiltrating immune cells in early‐stage pancreatic ductal adenocarcinoma
Source: IET Syst Biol. 2023 Feb 7;17(2):39–57. doi: 10.1049/syb2.12058 (PMC10116025; doi:10.1049/syb2.12058)
Supplement: Supplementary file 4 — Table S3 [file SYB2-17-39-s002.docx]

**Table S3** The members of LILR genes family

| Gene Symbol | Official Full Name |
| --- | --- |
| LILRA1 | leukocyte immunoglobulin like receptor A1 |
| LILRA2 | leukocyte immunoglobulin like receptor A2 |
| LILRA3 | leukocyte immunoglobulin like receptor A3 |
| LILRA4 | leukocyte immunoglobulin like receptor A4 |
| LILRA5 | leukocyte immunoglobulin like receptor A5 |
| LILRA6 | leukocyte immunoglobulin like receptor A6 |
| LILRB1 | leukocyte immunoglobulin like receptor B1 |
| LILRB2 | leukocyte immunoglobulin like receptor B2 |
| LILRB3 | leukocyte immunoglobulin like receptor B3 |
| LILRB4 | leukocyte immunoglobulin like receptor B4 |
| LILRB5 | leukocyte immunoglobulin like receptor B5 |
